# Adult *Fasciola* recovered from the biliary tree in a patient presenting with possible ascending cholangitis and acute acalculous cholecystitis

**DOI:** 10.1128/asmcr.00038-26

**Published:** 2026-05-04

**Authors:** Kaeli N. Bryant, Eileen Burd, Adam Barker, Blaine A. Mathison

**Affiliations:** 1 Department of Pathology, University of Utah 7060, Salt Lake City, Utah, USA; 2 Department of Pathology and Laboratory Medicine, Emory University 1371, Atlanta, Georgia, USA; 3 Infectious Diseases Operations, ARUP Laboratories 33294, Salt Lake City, Utah, USA; Rush University Medical Center, Chicago, Illinois, USA

**Keywords:** cholangitis, trematode, parasitology, *Fasciola*

## Abstract

**Background:**

Fascioliasis is a zoonotic trematode infection caused by members of the genus *Fasciola* (*F. hepatica* and *F. gigantica*) that localize to the hepatic biliary ducts in humans. Parasites are acquired after ingestion of freshwater plants, and fascioliasis has been reported on every inhabited continent. Typically, fascioliasis is diagnosed by serology or stool examination. This case reports the unusual finding of adult *Fasciola* organisms during an endoscopic retrograde cholangiopancreatography (ERCP).

**Case Summary:**

A 65-year-old woman who returned to the United States after traveling in Vietnam presented with abdominal pain, eosinophilia, and cholangitis. Filling defects/debris were detected in the common bile duct (CBD) upon imaging studies, so an ERCP was performed to investigate further. During the procedure, between three and four adult flukes were recovered from the biliary ducts and were later identified as *Fasciola* at a reference laboratory.

**Conclusion:**

The recovery of intact *Fasciola* during a surgical procedure is a highly unusual finding. It is crucial to accurately identify these organisms as there are numerous different species of liver flukes. Some flukes may resemble *Fasciola* at the egg or adult stage, such as *Fasciolopsis buski*. The treatment of *Fasciola* differs from that of all other liver flukes, as it is resistant to praziquantel and must be treated with triclabendazole, and improper treatment can lead to chronic complications.

## INTRODUCTION

Fascioliasis is a zoonotic disease caused by infection with *Fasciola hepatica* or *Fasciola gigantica. F. hepatica* is found nearly worldwide, and *F. gigantica* is localized to tropical and subtropical regions of Africa and Asia (1). Fascioliasis is characterized by an invasive (acute) phase and a biliary (chronic) phase (2). During the invasive disease phase, symptoms include local inflammation, abdominal pain, elevated transaminase levels, and eosinophilia (which is sustained through both phases) (3). The biliary disease phase symptoms include cholestasis, bile duct inflammation and fibrosis, and hepatic atrophy (3).

Fascioliasis is estimated to impact almost 17 million people globally and is recognized as a neglected tropical disease by the World Health Organization (4). Fascioliasis is typically diagnosed through serology or through the detection of eggs in stool (1, 5). Rarely, adult liver trematodes, or flukes, can be surgically removed from a host, but these organisms are found incidentally and are typically incomplete (6–9). In this report, we describe the unusual phenomenon of recovery of intact, adult *Fasciola* flukes from a patient in the United States.

## CASE PRESENTATION

The patient is a 65-year-old Vietnamese woman with a history of essential hypertension, who was admitted for evaluation of acute-onset upper abdominal pain with fever up to 103°F and transient hypotension and hypoxia thought to be a response to pain. Initial laboratory tests revealed leukocytosis (11.6 10E3/mcl; normal 4–10 10E3/mcl) and elevated liver function (alanine aminotransferase 128.4 units/L [normal = 7–52 units/L] and aspartate aminotransferase [AST 192 units/L, normal = 13–39 units/L]). Lab tests during hospitalization showed progressively rising bilirubin (2.3 mg/dL and then 3.8 mg/dL [normal 0.3–1.0 mg/dL]) and persistently elevated transaminases but normalized white blood cell count (7.5 → 5.0 10E3/mcl) and normal lactate. Imaging studies showed a dilated common bile duct (CBD) with mucosal hyperenhancement and an edematous gallbladder with a small amount of surrounding pericholecystic fluid and filling defects/debris within the CBD, highly suspicious for ascending cholangitis and acute acalculous cholecystitis. Also present were two hepatic masses and bilateral adnexal cystic masses that were present in a trauma CT from 2 years ago concerning for neoplasm but not followed-up on and now thought to be likely sequelae of chronic cholangitis since tumor markers (CA-125 8.8; CA 19-9, CEA, and AFP) are negative.

She underwent endoscopic retrograde cholangiopancreatography (ERCP) that resulted in the removal of three to four probable liver flukes from the biliary tree. A biliary stent was placed in the pancreatic duct with clearance of filling defects by the end of the procedure, followed by resolution of symptoms. She was treated with IV piperacillin-tazobactam and supportive care. Blood cultures remained negative throughout the admission. The patient reported last having been in Vietnam 2 years ago; while there, she ate vegetables (including uncooked greens) and fish, but no snails, and spent a lot of time at the beach. *Opisthorchis viverrini* is the most common liver fluke in the region of Vietnam where she lived, with *Fasciola* also being prevalent (10). She remembered that she had worms as a child in Vietnam.

**Fig 1:**
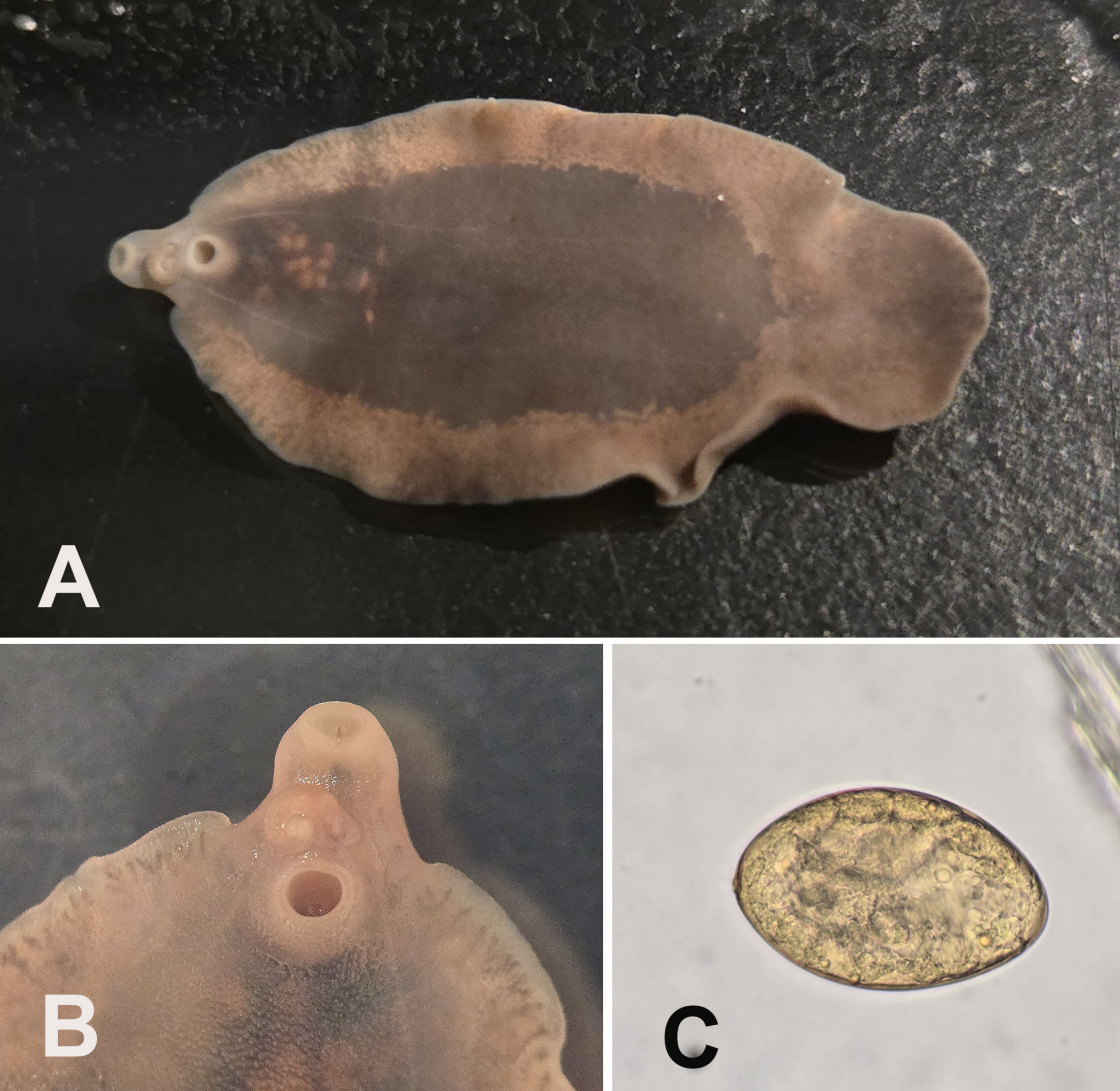
(**A**) The intact adult *Fasciola* that was surgically recovered during an ERCP; the fluke measured 3.2 cm long. (**B**) The oral sucker (top) and ventral sucker (bottom) of the adult *Fasciola*. (**C**) A *Fasciola hepatica* egg from domestic cattle measuring 140 µm long. This is a representative specimen for educational purposes, as no stool specimen was collected from the patient in this case report.

One of the flukes recovered in surgery was sent to a reference laboratory in 10% formalin for confirmatory identification. The fluke was flat, lanceolate, and measured 3.2 cm in length (Fig. 1A). It had prominently fleshy oral and ventral suckers (Fig. 1A and B). Based on the morphology, size, and having been extracted from a bile duct, it was identified as an adult *Fasciola* fluke.

## DISCUSSION

*F. hepatica* and *F. gigantica* are acquired following the ingestion of aquatic vegetation, such as watercress, dandelion greens, and water chestnuts (11). Additionally, *Fasciola* can also be acquired through the consumption of contaminated water, where the metacercariae (the infectious stage of fasciolids) have been dislodged from the vegetation (11). The definitive hosts for fasciolids are herbivorous ruminants, such as cattle or sheep (2). These herbivores excrete unembryonated eggs that embryonate in water and eventually hatch, releasing a first-stage larva (miracidium) (2). The miracidium then infects an appropriate host snail. After cycles of asexual reproduction in the snail host, cercariae are released, which encyst on aquatic vegetation, forming metacercariae, which are then consumed by ruminants, or incidentally, humans (2).

Within the definitive or incidental host, the metacercariae will excyst into an immature, or larval, fluke (2). This leads to the invasive phase of fascioliasis, where the larval fluke will travel from the intestine through the liver parenchyma to the biliary ducts (2). During the biliary phase of fascioliasis, the fluke matures into an adult and lays eggs, which are excreted by the host (2). Although hermaphroditic, the optimal form of reproduction is cross-fertilization between two flukes.

The World Health Organization has classified fascioliasis as a neglected tropical disease (4). Fascioliasis was previously believed to be a sporadic disease in humans; now, fascioliasis is estimated to impact almost 17 million people globally, with another 180 million people at risk (12–14). While many individuals remain asymptomatic, fascioliasis can cause severe disease, such as what this patient experienced (3). Treatment of fascioliasis differs from the treatment of all other liver flukes, as *F. hepatica* and *F. gigantica* are resistant to praziquantel and must be treated with triclabendazole (5, 15).

In this case report, the patient experienced many symptoms that are associated with fascioliasis. Eosinophilia is a characteristic symptom of fascioliasis and occurs in both phases of the disease (3). Persistently elevated transaminase levels occur because of the stress caused to the liver by the adult flukes. The patient also exhibited symptoms of cholangitis and cholecystitis, which were confirmed during surgery. She likely acquired the parasites after ingesting uncooked aquatic vegetables, which was a behavior described in the patient history.

Both *F. hepatica* and *F. gigantica* can be found in Vietnam, and hybrids between the two species are becoming increasingly common (1, 16–19). The fluke in this case was slightly outside the standard published range for *F. hepatica* (up to 3.0 cm), the body was subparallel, and the shoulders at the base of the oral cone were not prominent (Fig. 1A and B) (20). These features would generally support an identification of *F. gigantica*; however, given the plasticity of adult flukes, possible effects of formalin fixation, and the presence of *F. hepatica-F. gigantica* hybrids in Vietnam, the identification was restricted to the genus level (19). While the two species can be differentiated by egg morphology, the expression of eggs from the adult fluke was unsuccessful, and eggs were also not found in the transport medium.

The two main methods of diagnosing fascioliasis are serologic testing and wet examination of stool for eggs (1, 5). Fasciolids produce several proteins that stimulate antibody production early in infection, so a diagnosis of fascioliasis can be made with serology before eggs can be detected in stool (3 to 4 months after exposure) (1, 2). There is currently no FDA-cleared serologic assay for detection of fascioliasis. Laboratory-developed enzyme-linked immunosorbent assays (ELISAs) with high specificity and sensitivity have been described for the detection of antibodies against saposin-like protein 2 antigen, cathepsin L, and glutathione S-transferase (21–25). These tests were developed using *F. hepatica* recombinant proteins, and their cross-reactivity for *F. gigantica* is mostly undetermined. However, it is known that these ELISAs can be cross-reactive for antibodies produced against other helminths, including *Echinococcus* (26).

Microscopically, *F. hepatica* eggs are 130–140 µm long and 63–90 µm wide, as shown in Fig. 1C (egg image not from this case) (2). The eggs have a thin, defined border and a clear, inconspicuous operculum at one pole (Fig. 1C). The eggs of *F. hepatica* are nearly identical to those of the large intestinal fluke, *Fasciolopsis buski*, and are very similar to those of other intestinal flukes such as *Gastrodiscoides hominis* and some *Echinostoma* species. Eggs of *F. gigantica* are larger, measuring 160–190 µm long by 70–90 µm wide. Notably, the detection of eggs in stool may be due to spurious passage after consumption of infected animal liver, so it is important to confirm diagnosis with multiple stool specimens (11). In this case, a wet stool examination was not ordered for the patient as the recovery of flukes during surgery was likely considered to be sufficient for diagnosis of fascioliasis.

Rarely, adult flukes can be detected or directly recovered from the biliary ducts during a surgical procedure, such as an ERCP (6–9, 27). Surgery is not a recommended form of intervention if fascioliasis is suspected or diagnosed; however, these organisms can be incidentally discovered during surgical management of symptoms caused by fascioliasis. In the case of our patient, an ERCP was performed to manage bile duct blockage. Since the adult flukes were completely removed during surgery, the clinical team did not treat the patient with triclabendazole, which is the typical treatment for fascioliasis. However, administration of triclabendazole would treat flukes that may have incidentally migrated to other parts of the body. The patient reported complete resolution of her symptoms at a follow-up visit 1 month after the ERCP, suggesting that the fascioliasis may have resolved.

It is critical to be able to distinguish *Fasciola* from other liver flukes at both the egg and adult stages. While an adult liver fluke is a very rare specimen to receive in the clinical microbiology lab, it is crucial that the lab accurately identifies the organism due to differences in how the patient will be treated for the infection.
